# Supplementary material for: Analog in-memory computing attention mechanism for fast and energy-efficient large language models
Source: Nat Comput Sci. 2025 Sep 8;5(9):813–24. doi: 10.1038/s43588-025-00854-1 (PMC12457188; doi:10.1038/s43588-025-00854-1)
Supplement: Supplementary file 1 — Supplementary Text/Discussion, Figs. 1–7 and Algorithm 1. [file 43588_2025_854_MOESM1_ESM.pdf]

nature computational science

Supplementary information

https://doi.org/10.1038/s43588-025-00854-1

Analog in-memory computing attention mechanism for fast and energy-efficient large language models

In the format provided by the authors and unedited

Contents

1 CMOS layout **1**
1.1 HardSigmoid charge-to-pulse converter 1
1.2 Signed charge-to-pulse converter 3

2 Adaptation Algorithm **5**
2.1 Pseudo-code 5
2.2 Generalization to Different Nonlinear Functions 6

3 Effect of capacitor's leakage **6**
3.1 Experiment on different decay factor values 6
3.2 Theoretical study of attention scores' decay and link with ALiBi positional embeddings 7

4 Mapping Transformer Operations to 3D OSFET In-Memory Computing Layers **8**
4.1 System level implementation 9
4.2 Thermal Management 9

1 CMOS layout

We design a custom CMOS layout of the proposed gain cell and charge-to-pulse circuits. In this study, the circuit simulations were done in TSMC 28 nm silicon CMOS technology. We used this conventional design style as a proof of concept to demonstrate the capacity of our gain cells-based architecture to perform the attention mechanism. However, CMOS gain cells lead to relatively large area footprint, primarily due to Metal-Oxide-Metal (MOM) capacitors which must be relatively large due to their high leakages. Our layout results show that each cell has a dimensions of $3.9\,\mu\text{m}\times4.9\,\mu\text{m}$, resulting in an area of $0.08\,\text{mm}^2$ per 64×64 array, or $1.28\,\text{mm}^2$ for one entire attention head (16 sub-tiles). In comparison, the HardSigmoid charge-to-pulse circuitry and its signed variant occupy an area of $0.01\,\text{mm}^2$ and $0.02\,\text{mm}^2$ per attention head, respectively. The Layout of the gain cells storing V values and computing $\phi(S)\cdot V$ is shown in shown in Supplementary Figure 1. Note that the Layout of the gain cells storing K has transposed World Lines (WL) and Bit Lines (BL).

1.1 HardSigmoid charge-to-pulse converter

In this section, we provide additional information on the working principle for the HardSigmoid charge-to-pulse circuit block. This charge-to-pulse circuit operates in three distinct phases: sampling, discharge, and reset. During the sampling phase, input pulses are applied to the first gain cell array, and the currents generated by the cells are integrated by a capacitor (C_2) in the charge-to-pulse circuit. This capacitor also utilizes the wire capacitance of the word line. In the discharge phase, the voltage of the capacitor C_2 is discharged with a constant current controlled by the bias voltage V_b. However it is important to note that the system employs an energy saving scheme by checking the voltage on the integrating capacitor V_{cap} and only preforming the

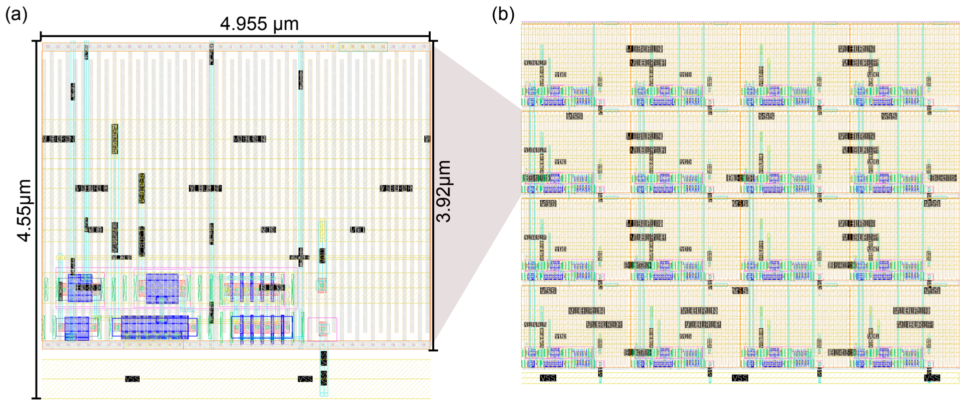

Supplementary Figure 1 Pure CMOS Layout of a gain cell array. (a) Layout of a single cell storing V values on a Metal-on-Metal (MoM) capacitor and computing $\phi(S) \cdot V$ in-memory. (b) 4×4 array Layout. Note that apart of the bottom row the hight scales with $3.92\,\mu$m

discharge in case the voltage is positive. An inverter acts as a simple comparator, triggering a pulse of variable width. Finally, in the reset phase, the bit line is reset to the initial bit line voltage to prepare for a new inference step.

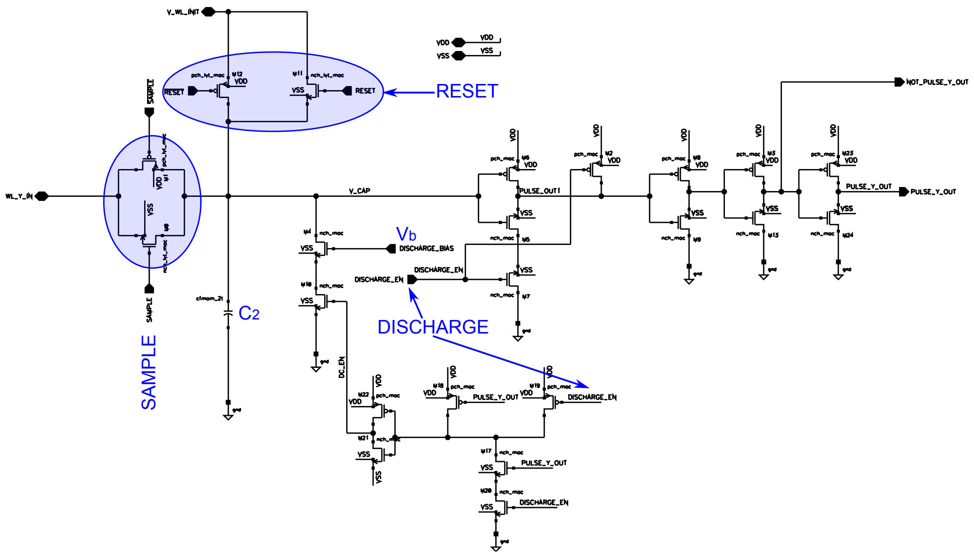

Supplementary Figure 2 HardSigmoid Charge-to-Pulse Converter Circuit. CMOS schematic of the charge-to-pulse circuit implementing the HardSigmoid function. The circuit operates in three phases—sampling, discharge, and reset—integrating current onto a capacitor and generating an output pulse whose width encodes the input magnitude. Energy-efficient operation is achieved via a conditional discharge mechanism

1.2 Signed charge-to-pulse converter

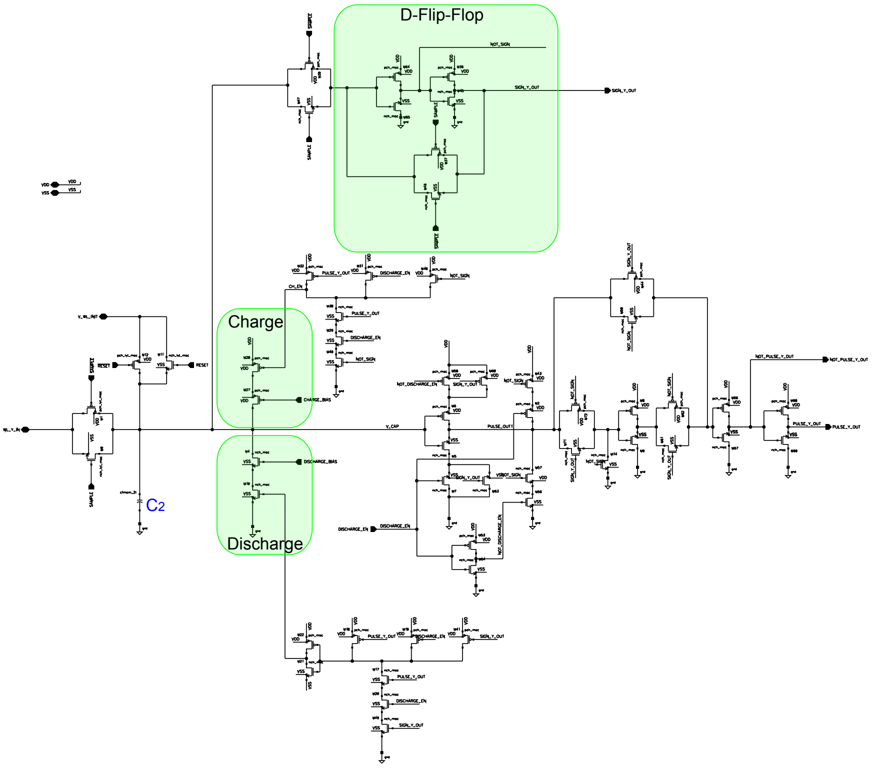

Supplementary Figure 3 Signed Charge-to-Pulse Converter Circuit. CMOS schematic of the charge-to-pulse converter designed to handle signed Multipy-Accumulate (MAC) results. In contrast to the HardSigmoid variant, this circuit introduces a bidirectional charging mechanism, enabling both charge-up and charge-down paths. A D flip-flop captures the polarity of the voltage on the integrating capacitor at the end of the sampling phase, determining the subsequent discharge or charge operation. The pulse generation block is adapted to ensure consistent high-active output pulses while preserving sign information.

To implement a signed charge to pulse circuit, the main difference from the circuit in the Supplementary section "HardSigmoid charge-to-pulse converter" is the addition of a charge-up path and a D Flip-Flop. This serves the following purpose: at the end of the sampling stage, the D Flip-Flop captures the polarity of the voltage on the capacitor and stores it for subsequent operations. This stored sign determines whether a charge or discharge is applied to the capacitor voltage. The pulse-forming circuit is now slightly more complex to ensure consistent, high-active output pulses. Ultimately, the circuit outputs both the sign and the output pulses. Supplementary Figure 3 shows the circuit schematics. The two distinct charge up and charge down behaviours given a certain sign are displayed in Supplementary Figure 4.

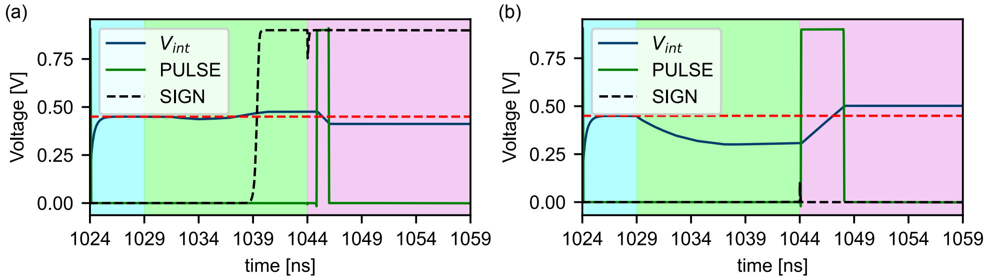

Supplementary Figure 4 Transient behavior of the output readout circuit. Example of a MAC result with (a) a positive sign and (b) a negative sign, illustrating distinct charge dynamics for each case.

2 Adaptation Algorithm

2.1 Pseudo-code

Supplementary Algorithm 1 Pseudo-code for the adaptation algorithm used to map the nonlinear model to the linear model.

g^L ▷ Linear model's modules
g^{NL} ▷ Nonlinear model's modules
$INPUT_0 \leftarrow SAMPLE$ ▷ Get text from the dataset
$\epsilon \in]0,1[$ ▷ Error threshold
$\gamma \in]0,1]$ ▷ Measure rate
$\Gamma \in]0,1]$ ▷ Adaptation rate
$ERROR \leftarrow 1$
for i in MODULES INDEXES **do** ▷ Inference on the linear model
    $INPUT_i \leftarrow g_i^L\left(INPUT_{i-1}\right)$
end for
for i in SCALING MODULES INDEXES **do** ▷ Measure the linear model statistics
    $x \leftarrow INPUT_i$
    $y \leftarrow a_i^L x + b_i^L$
    $\sigma_i^L \leftarrow \sqrt{\frac{1}{n}\sum_j^n \left(y_j - |y|\right)^2}$
    $\mu_i^L \leftarrow |y|$
end for
while $ERROR > 0$ **do** ▷ Adaptation loop
    $ERROR \leftarrow 0$
    for i in MODULES INDEXES **do** ▷ Inference on the nonlinear model
        $INPUT_i \leftarrow g_i^{NL}\left(INPUT_{i-1}\right)$
    end for
    for i in SCALING MODULES INDEXES **do** ▷ Measure statistics and adapt scaling
        $x \leftarrow INPUT_i$
        $y \leftarrow a_i^{NL} x + b_i^{NL}$
        $\sigma_i^{NL} \leftarrow \gamma\sqrt{\frac{1}{n}\sum_j^n \left(y_j - |y|\right)^2} + (1-\gamma)\,\sigma_i^{NL}$
        $\mu_i^{NL} \leftarrow \gamma|y| + (1-\gamma)\,\mu_i^{NL}$
        if $|\sigma_i^{NL} - \sigma_i^L| > \epsilon$ **then**
            $ERROR \leftarrow ERROR + 1$
            $a_i^{NL} \leftarrow \Gamma a_i^{NL}\frac{\sigma_i^L}{\sigma_i^{NL}} + (1-\Gamma)\,a_i^{NL}$
        end if
        if $|\mu_i^{NL} - \mu_i^L| > \epsilon$ **then**
            $ERROR \leftarrow ERROR + 1$
            $b_i^{NL} \leftarrow \Gamma\left(b_i^{NL} + \mu_i^L - \mu_i^{NL}\right) + (1-\Gamma)\,b_i^{NL}$
        end if
    end for
end while

2.2 Generalization to Different Nonlinear Functions

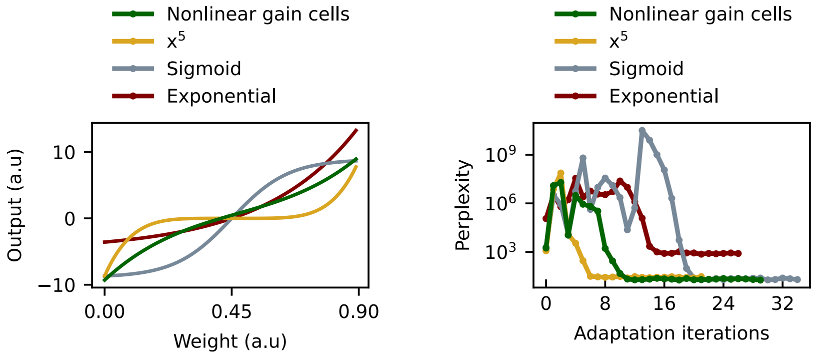

Supplementary Figure 5 Adaptation mechanism evaluated with different nonlinear functions. (a) Different nonlinear functions tested in place of the gain cells nonlinearities. (b) Evolution of perplexity (lower the better) during the adaptation algorithm when our attention model is implemented with different nonlinearities applied on the stored keys and values.

In this experiment, we evaluate the capacity of our adaptation algorithm to generalize to other nonlinearities than the one modelling the gain cells (see Supplementary Figure 5). We perform the dot-products of the attention mechanism with different nonlinearities applied to the stored keys and values. The different functions tested are: $f(x) = \alpha (x - \beta)^5$, $f(x) = \alpha sigmoid (10 (x - \beta))$, and $f(x) = \alpha e^{3(x-\beta)}$, with α and β chosen to yield to similar ranges for the different functions.

We see that our adaptation algorithm manages to reduce the perplexity drastically, except for the exponential function. The high asymmetry of the exponential function prevents the network to yield good accuracy. Thus, the adaptation algorithm manages to reduce the perplexity for functions which are anti-symmetric even if they are highly nonlinear, such as x^5 (perplexity=29) or *sigmoid* (perplexity=21).

3 Effect of capacitor's leakage

In this section, we study both from a theoretical and an experimental perspective the effect of the decay induced by the leakage of Gain Cells' capacitors on the attention computation.

3.1 Experiment on different decay factor values

The effect of decay on the computation varies depending on the global latency of the system Δt and on the retention times τ of the Gain Cells as shown in equation 5 in the main text. To study the effect of decay in multiple configurations, in Supplementary Figure 6 we show the evolution of perplexity when the value of the decay factor $\frac{\Delta t}{\tau}$ is swapped for the trained hardware model. The lowest perplexity was found for $\frac{\Delta t}{\tau} = 10^{-4}$, which comes from the fact that the model was trained with a similar decay

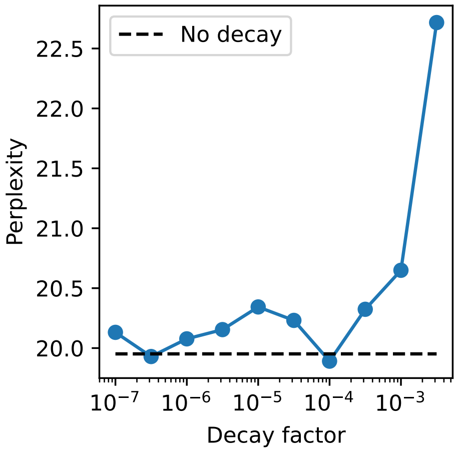

Supplementary Figure 6 Effect of decay factor on the evaluation perplexity. The hardware model was trained with a decay factor of 1.6×10^{-4}. The horizontal axis is on a $\log_{10}$ scale.

factor of 1.6×10^{-4}. The perplexity starts increasing more significantly for $\frac{\Delta t}{\tau} > 10^{-3}$, but training the model with larger decay factors could potentially allow the model to reach better evaluation results with larger decay factors.

3.2 Theoretical study of attention scores' decay and link with ALiBi positional embeddings

ALiBi [1] is a method to inject positional knowledge using relative decays in the attention scores. In this section, we study the mathematical similarities between the decay caused by capacitors' leakage with ALiBi. The self-attention with ALiBi positional embedding is

$$
\begin{aligned}
A_i &= softmax\left(Q_i \cdot K^T - h\left[-\left(i-1\right), ..., -2, -1, 0\right]\right) \cdot V \\
&= softmax\left(Q_i \cdot K^T - \max\left(0, i - \mathbf{j}\right) h\right)\right) \cdot V \\
&= \frac{e^{Q_i \cdot K_j^T - \max(0, i-j)h}}{\sum_j e^{Q_i \cdot K_j^T - \max(0,i-j)h}} \cdot V \\
&= e^{-\max(0,i-j)h} \frac{e^{Q_i \cdot K_j^T}}{\sum_j e^{Q_i \cdot K_j^T - \max(0,i-j)h}} \cdot V
\end{aligned} \quad . \tag{1}
$$

Since the Gain Cells' leakage induces decays α_i in the stored keys and values, the attention with Gain Cells is

$$
A_i = \phi\left(Q_i \cdot k^T \alpha_i\right) \cdot V \alpha_i . \tag{2}
$$

In the non-saturated regime, when the attention scores $S_i = Q_i \cdot k^T \alpha_i$ are superior to 0 and inferior to S_{sat}, we can simplify the equation as

$$\begin{aligned} A_i &= \left(Q_i \cdot k^T \alpha_i\right) \cdot V \alpha_i \\ &= \left(\left(Q_i \cdot k^T\right) \alpha_i^2\right) \cdot V \end{aligned} \tag{3}$$

with $\alpha_i = e^{-\frac{\Delta t}{\tau} max(0,i-j)}$. Both attention scores are therefore weighted by an exponential decay depending on the relative positions of the token. These relative decay components for the ALiBi attention $\Delta_{ALiBi_{i,j}}$ and from the Gain Cell attention $\Delta_{GainCells_{i,j}}$ are

$$\begin{aligned} \Delta_{ALiBi_{i,j}} &= e^{-\max(0,i-j)h} \\ \Delta_{GainCells_{i,j}} &= \alpha_i^2 \\ &= e^{-2\frac{\Delta t}{\tau} max(0,i-j)} \end{aligned} . \tag{4}$$

Let us now compare the decay amplitude of the two attentions. We start from the hypothesis $\Delta_{GainCells_{i,j}} \geq \Delta_{ALiBi_{i,j}}$:

$$\begin{aligned} &\Delta_{GainCells_{i,j}} \geq \Delta_{ALiBi_{i,j}} \\ &\Leftrightarrow e^{-2\frac{\Delta t}{\tau}\max(0,i-j)} \geq e^{-\max(0,i-j)h} \\ &\Leftrightarrow \frac{\Delta t}{\tau} \leq \frac{h}{2} \end{aligned} . \tag{5}$$

This equation shows that $\Delta_{GainCells_{i,j}} \geq \Delta_{ALiBi_{i,j}}$, which means that the decay is equal or slower on the Gain Cells attention than on the ALiBi attention if and only if $\frac{\Delta t}{\tau} \leq \frac{h}{2}$. In the article [1], the largest slope (faster decay) h used is $h = \frac{1}{2}$ and the smallest (slowest decay) is $h = \frac{1}{2^8} \simeq 4 \times 10^{-3}$. In comparison, our decay factor is $\frac{\Delta t}{\tau} = 1.6 \times 10^{-4}$. In conclusion, this study shows that even faster capacitive decays could be tolerated and could naturally inject relative token position information, as proposed in the ALiBi embeddings.

4 Mapping Transformer Operations to 3D OSFET In-Memory Computing Layers

Reducing the area footprint of memory arrays storing K-V pairs is fundamental to implement Large Language Models at scale. To reduce the area footprint, gain cells can be vertically stacked in three dimensions [2]. In fact, commercial DRAM technologies have already demonstrated the feasibility of stacking more than twelve layers [3]. While a 3D architecture can significantly improve throughput, it also introduces challenges related to heat dissipation, which is a critical concern in such densely integrated systems. In this section, we propose a hardware design that efficiently implements multiple Transformer layers and attention heads using 3D-stacked OSFET-based in-memory computing arrays. We also analyze the thermal implications of this architecture. In this section, we propose a hardware design that efficiently implements multiple

Transformer layers and attention heads using 3D-stacked OSFET-based In-Memory-Computing (IMC) arrays, where stacking minimizes area footprint and sequential physical layer activation helps manage thermal constraints.

4.1 System level implementation

In Supplementary Figure 7, we show how Transformers can be implemented at the system level with attention heads mapped to 3D-stacked gain cells memory. Supplementary Figure 7 (a) illustrates an example Transformer model featuring 3 layers and 3 parallel attention heads. The different attention heads are mapped onto separated parallel hardware blocks working in parallel, as represented in Supplementary Figure 7 (b). Each block comprises a single base silicon CMOS wafer integrating control logic, nonlinear activation functions, readout circuitry, and digital arithmetic units. To perform the dot-products and to store the keys and values, gain cells of OSFET technology are stacked vertically. Each vertical stack maps a different Transformer layer. In Supplementary Figure 7 (b), each sub-block (e.g., OSFET K1.1) represents one attention head for one Transformer layer, and is therefore implemented by multiple sub-tiles, as depicted in the Results section "Full Attention Head Hardware Implementation". To scale beyond the available number of 3D memory stacks, additional Transformer layers can be mapped onto separate hardware blocks. The choice of mapping Transformers' layers vertically onto different stacks is motivated by the sequential execution of Transformer layers. The advantages are twofold: (1) each CMOS wafer can serve multiple stacked memory arrays without requiring replication, and (2) only one layer of the stack is active during attention inference, which helps reduce thermal buildup. Using existing 3D technologies, this design could reduce the area footprint of in-memory computing arrays by one order of magnitude compared to 2D designs. In this study, we focus exclusively on the computation of the attention mechanism. However, the proposed architecture is also compatible with IMC arrays capable of performing the linear layers required for a complete network. These linear layers could be integrated in a 3D-stacked configuration using non-volatile memory technologies, as demonstrated in [4].

4.2 Thermal Management

To assess the risk of thermal buildup, we estimate the power dissipation of the main heat sources in the system: the silicon CMOS base layers and the gain cell crossbar arrays. According to our design (Fig. 3(b)) and to our energy estimation simulations in the Results section "Energy Consumption and Latency", the average power density dissipated by the CMOS part during a 65 ns attention inference step is $27\,\mathrm{W/cm^2}$. Because different stacks in the vertical structure implement different Transformer layers, only one gain cell stack is active at any given time. According to our simulations and design, the gain cells arrays dissipate in average a power density of $10\,\mathrm{W/cm^2}$ during attention inference. Thus, the maximum combined power density of the vertical stack is approximately $37\,\mathrm{W/cm^2}$, which is comparable to that of 3D-stacked High Bandwidth Memory (HBM)s [3]. Notably, [3] demonstrated that such HBM structures can operate reliably at up to $45\,\mathrm{W/cm^2}$. Moreover, hardware attention blocks

will not be active continuously; during other processing stages—such as execution of Feedforward Neural Networks (FNNs)—these units will cool down, further mitigating thermal stress. In conclusion, our 3D design significantly reduces area footprint while maintaining thermal safety. The sequential activation of stacks and moderate power dissipation contribute to a low risk of thermal buildup.

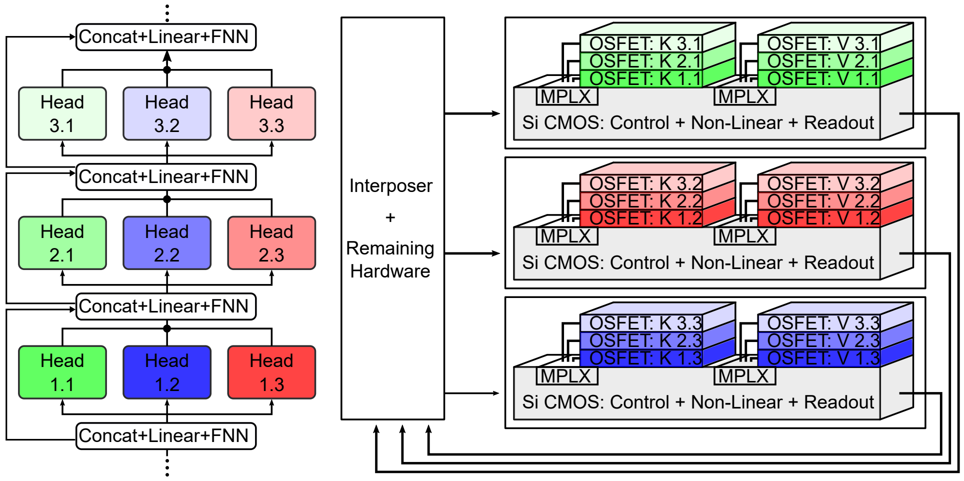

Supplementary Figure 7 3D-stacked gain cells-based Transformer design. (a) Simplified schematic of a Transformer architecture, illustrating 3 layers, each with 3 parallel attention heads. (b) Proposed system-level architecture mapping Transformer attention heads onto hardware using 3D-stacked OSFET-based In-Memory Computing arrays. Each OSFET stack stores the keys (K) and values (V) corresponding to a different Transformer layer. A single underlying silicon CMOS chip performs non-linear operations, control logic, and readout.

References

[1] Press, O., Smith, N.A., Lewis, M.: Train short, test long: Attention with linear biases enables input length extrapolation. In: ICLR (2022). https://openreview.net/forum?id=R8sQPpGCv0

[2] Ye, H., Gomez, J., Chakraborty, W., Spetalnick, S., Dutta, S., Ni, K., Raychowdhury, A., Datta, S.: Double-Gate W-Doped Amorphous Indium Oxide Transistors for Monolithic 3D Capacitorless Gain Cell eDRAM. In: 2020 IEEE International Electron Devices Meeting (IEDM), pp. 28–312834 (2020). https://doi.org/10.1109/IEDM13553.2020.9371981 . https://ieeexplore.ieee.org/document/9371981/?arnumber=9371981

[3] Son, K., Park, J., Kim, S., Sim, B., Kim, K., Choi, S., Kim, H., Kim, J.: Thermal Analysis of High Bandwidth Memory (HBM)-GPU Module considering Power Consumption. In: 2023 IEEE Electrical Design of Advanced Packaging and Systems (EDAPS), pp. 1–3 (2023). https://doi.org/10.1109/EDAPS58880.2023.10468315 . https://ieeexplore.ieee.org/document/10468315/

[4] Büchel, J., Vasilopoulos, A., Simon, W.A., Boybat, I., Tsai, H., Burr, G.W., Castro, H., Filipiak, B., Le Gallo, M., Rahimi, A., Narayanan, V., Sebastian, A.: Efficient scaling of large language models with mixture of experts and 3D analog in-memory computing. Nature Computational Science, 1–14 (2025) https://doi.org/10.1038/s43588-024-00753-x
